# Decomposing economic disparities in risky sexual behaviors among people who inject drugs in Tehran: Blinder-Oaxaca decomposition analysis

**DOI:** 10.4178/epih.e2017049

**Published:** 2017-11-05

**Authors:** Mehdi Noroozi, Hamid Sharifi, Alireza Noroozi, Fatemah Rezaei, Mohammad Rafi Bazrafshan, Bahram Armoon

**Affiliations:** 1 Social Determinants of Health Research Center, University of Social Welfare and Rehabilitation Sciences, Tehran, Iran; 2 HIV/STI Surveillance Research Center, WHO Collaborating Center for HIV Surveillance, Institute for Future Studies in Health, Kerman University of Medical Sciences, Kerman, Iran; 3 Iranian National Center for Addiction Studies, Tehran University of Medical Sciences, Tehran, Iran; 4 Department of Neuroscience and Addiction Studies, School of Advanced Technologies in Medicine, Tehran University of Medical Sciences, Tehran, Iran; 5 Department of Social Medicine, School of Medicine, Jahrom University of Medical Sciences, Jahrom, Iran; 6 Department of Nursing, School of Nursing, Larestan University of Medical Sciences, Larestan, Iran; 7 Student Research Committee, School of Health, Shahid Beheshti University of Medical Sciences, Tehran, Iran

**Keywords:** Economics, Sexual behavior, Drug users, Cross-sectional studies, Iran

## Abstract

**OBJECTIVES:**

To our knowledge, no previous study has systematically assessed the role of economic status in risky sexual behavior among people who inject drugs (PWID) in Iran. In this study, we used Blinder-Oaxaca (BO) decomposition to explore the contribution of economic status to inequality in unprotected sex among PWID in Tehran and to decompose it into its determinants.

**METHODS:**

Behavioral surveys among PWID were conducted in Tehran, the capital city of Iran, from November 2016 to April 2017. We employed a cross-sectional design and snowball sampling methodology. We constructed the asset index (weighted by the first principal component analysis factor) using socioeconomic data and then divided the variable into 3 tertiles. We used the BO method to decompose the economic inequality in unprotected sex.

**RESULTS:**

Of the 520 recruited individuals, 20 were missing data for variables used to define their economic status, and were therefore excluded from the analysis. Not having access to harm reduction programs was the largest factor contributing to the economic disparity in unprotected sex, accounting for 5.5 percentage points of the 21.4% discrepancy. Of the unadjusted total economic disparity in unprotected sex, 52% was unexplained by observable characteristics included in the regression model. The difference in the prevalence of unprotected sex between the high-income and low-income groups was 25%.

**CONCLUSIONS:**

Increasing needle syringe program coverage and improving human immunodeficiency virus (HIV) knowledge are essential for efforts to eliminate inequalities in HIV risk behaviors among PWID.

## INTRODUCTION

It is estimated that 15.9 million (range, 11.0 to 21.2 million) people use drugs through injection globally [1], of whom 1.65 million are infected with human immunodeficiency virus (HIV) [2]. HIV infection among people who inject drugs (PWID) has been reported in more than 120 countries, and in many countries in Europe, Asia, and particularly in the Middle East, unsafe injection is the main mode of HIV transmission [1]. PWID are at increased risk of HIV infection due to both high-risk injecting and sexual practices [3]. In Iran, the PWID population size is estimated to be 170,000 to 230,000 people [4-6]. Risky sexual behaviors, such as having multiple partners, sex work, and unprotected intercourse, are common among PWID in Iran [7,8]. Unprotected sexual contacts in Iran were reported among more than 60% of PWID in their last sexual encounter with a commercial or casual partner [7]. This pattern of behavior allows HIV infection to spread within both injection and sexual networks [9,10]. A recent study of female partners of PWID in 3 cities in Iran showed the HIV prevalence to be as high as 2.8% among those who themselves did not inject drugs [7]. Many studies have assessed factors associated with risky sexual behavior among PWID [7,11,12]. Risky sexual behavior was significantly associated with HIV infection, the type of drug used, and the category of the partners [13-15]. Economic disparities in HIV-related high-risk behaviors among PWID remain [16]. Previous studies in some countries have shown risky sexual behaviors to be more prevalent in low economic status communities [17,18]. Low economic status and economic disparities may explain the differences in risky sexual behavior that cannot be explained by other individual or structural factors [18]. Lack of financial support and unstable economic status have been shown to be associated with high-risk behaviors, such as unprotected sex and sex work [19]. Previous studies have found economic disparities to play a role in HIV risk behavior, after adjusting multiple regression models for various observable characteristics, such as demographics and health status [18]. However, previous studies have not measured the degree to which these characteristics contribute to disparities. This knowledge is important for establishing and developing more effective HIV prevention programs. Moreover, the determinants of socioeconomic inequalities in access to and utilization of needle syringe programs (NSPs) have not been empirically investigated. To our knowledge, no previous study has systematically assessed the role of economic status in risky sexual behavior among PWID in Iran. In this study, we used Blinder-Oaxaca (BO) decomposition to explore the contribution of economic status to inequality in unprotected sex among PWID in Tehran and to decompose it into its determinants.

## MATERIALS AND METHODS

Behavioral surveys among PWID were conducted in Tehran, the capital city of Iran, from November 2016 to April 2017. We employed a cross-sectional design and snowball sampling methodology. Eligible individuals were aged 18 years or older, reported injection drug use in the past month, were able to speak and comprehend Farsi enough to respond to the survey questions, and were able to provide informed consent. Study participants were recruited from street locations by snowball sampling. These participants were then given the opportunity to invite their peers to participate in the study. The participants recruited through snowball sampling also received coupons to distribute to their peers. The study procedures were reviewed and approved by the Ethical Committee of the University of Social Welfare and Rehabilitation Sciences, Tehran, Iran. All data remained anonymous and confidential. The survey questionnaire used was a standardized behavioral questionnaire for PWID published by Family Health International [16]. The survey questionnaire included questions on socio-demographic characteristics, drug use and unprotected sex, access to harm reduction services, and HIV knowledge (low vs. high). The demographic variables assessed included age, marital status (0, married or cohabitating; 1, unmarried), highest level of education (0, less than high school; 1, high school diploma), homelessness in the past year (0, no; 1, yes), methamphetamine use (0, no; 1, yes) and history of incarceration (0, no; 1, yes ). HIV/acquired immune deficiency syndrome (AIDS) knowledge was measured with a 10-item set of questions covering basic knowledge of HIV/AIDS, to which participants could answer ‘yes,’ ‘no,’ or ‘don’t know.’

The outcome of interest was unprotected sex with any partner. Respondents were asked whether they had sex without a condom during last 6 months (0, no; 1, yes).

Our main covariate was economic status, which was assessed using principal component analysis (PCA). The data about socioeconomic status were gathered by a questionnaire that was developed in another study, entitled “Socio-economic status in Iran: a study of measurement index” [20]. The variables related to socioeconomic status in this questionnaire were educational status (high school or less vs. diploma or higher), participants’ employment status (employed vs. unemployed), income status (monthly income), and housing (homelessness vs. stable housing), as well as items of convenience, such as owning their own car. Other covariates in our analysis included demographic and behavioral characteristics, such as age (years), age of first drug use (≥ 25 years, 1; < 25 years, 0), age of first engaging in unprotected sex (≥ 25 years, 1; < 25 years, 0), age of first drug injection (≥ 25 years, 1; < 25 years, 0), HIV testing history, and marital status (single vs. married). HIV testing history was ascertained by the yes/no question ‘Have you ever had an HIV test?’ Having ever been tested was defined as having been tested for HIV and receiving the results at least once before the survey. All other behavioral questions referred to the 3 months prior to the interview.

We constructed the asset index (weighted by the first PCA factor) using socioeconomic data, and then divided the variable into 3 tertiles. The sum of score for the questionnaire was 48. The first tertile was considered to be the high-income group and the third tertile to be the low-income group. We calculated the prevalence of unprotected sex among PWID with low and high incomes. The chi-square test was used to compare HIV-related high-risk sexual behaviors between the 2 PWID subgroups. We then used the BO method to decompose the economic inequality in unprotected sex. This involved decomposing the observed high-low economic gaps in the prevalence of unprotected sex into 2 components: composition and response effects. Composition effects represent the contribution of economic inequalities to unprotected sex due to economic differences in the distributions of observable HIV risk factors between the high and low groups (i.e., socio-demographic characteristics). Response effects reflect the contribution of economic inequalities in unprotected sex to economic differences in the effects of measured factors, as well as unmeasured factors not included in the model. To perform the decomposition, we used a logistic regression model with independent variables in each income group to determine the regression coefficient (β) of each variable as the main effect and its interaction with the other independent variables. This method was based on 2 regression models, fitted separately for the 2 subgroups (i.e., the high-income and low-income groups). The analyses were performed in Stata version 11 (StataCorp., College Station, TX, USA) with an available Oaxaca package that supported the nonlinear decomposition for binary dependent variables proposed by Yun [19].

The BO decomposition method was introduced first by Blinder [21] and Oaxaca [22] to examine racial/gender discrimination in the labor market. The core idea is to explain the distribution of the outcome variable in question by a set of variables that vary systematically with socioeconomic status. The BO decomposition technique is especially useful for identifying and quantifying the separate contributions of group differences in measurable characteristics, such as education, experience, marital status, and geographical location, to racial and sex gaps in outcomes [23]. The aim of BO decomposition is to explain how much of the difference in mean outcomes between 2 groups is due to group differences in the levels of the explanatory variables, and how much is due to differences in the magnitude of the regression coefficients [23].

(1) YU-YL=[∑i=1NLF(XiLβH)NL-∑i=1NHF(XiHβH)NH]+[∑i=1NLF(XiLβL)NL-∑i=1NLF(XiLβH)NL]

In equation (1), N refers to the sample size for the high-income and low-income groups. The first term in brackets shows the part of the risky sexual behavior gap that is attributable to differences in the distribution of characteristics (the explained component or endowments effect), and the second term represents the portion of the risky sexual behavior gap that is due to differences in the effects of these characteristics on risky sexual behaviors (the unexplained component or coefficient effect).

## RESULTS

Out of the 520 recruited individuals, 20 were missing data for variables used to define their economic status, and were therefore excluded from the analysis. The study participants’ age ranged from 19 to 67 years, with a median of 32 years (interquartile range, 24 to 38 years) (Table 1). Only 38.6% were married at the time of the study, and 36.4% had completed fewer than 6 years of education. Of the participants, 60.8% reported a monthly income of 150 US dollars or more. Fifty-two percent had started drug use before the age of 25 years, and unprotected sex in the past 6 months was reported by 40.2% (Table 1).

Table 2 shows coefficient estimates (odds ratios [ORs]) between the income groups in multivariable logistic regression models. In the high-income group, PWID who were homeless (OR, 1.48; p= 0.03), had low HIV knowledge (OR, 1.60; p= 0.04), and had no access to harm reduction programs (OR, 2.70; p= 0.01) were more likely to have unprotected sex (Table 2). In the low-income group, PWID who were homeless (OR, 2.91; p=0.03), had low HIV knowledge (OR, 2.85; p= 0.02), and had no access to harm reduction programs (OR, 1.30; p= 0.30) were more likely to report unprotected sex. We found that the associations between a covariate and the dependent variable (unprotected sex) varied across income groups. For instance, the coefficient (OR) estimates for age of first drug use (1.12 vs. 1.42), HIV knowledge (1.60 vs. 2.85), and lack of access to harm reduction programs (2.70 vs. 1.30) were considerably different between the high-income and low-income groups.

Table 3 presents a detailed decomposition of the disparity by the differences in the estimated coefficient (i.e., ORs) for each covariate in the logistic regression models summarized in Table 2. For instance, the estimated coefficient of not having access to harm reduction programs was lower in the low-income group (OR, 1.30 in Table 2) than in the high-income group (OR, 2.70), which led to an increase of the low-high disparity by 5.5 percentage points (the largest ‘share’ of the 21.4% discrepancy). The ‘share’ of the constant term indicates that out of the unadjusted total low–high disparity in unprotected sex, 53% was still unexplained by the observable characteristics included in the regression model. The difference in the prevalence of unprotected sex between the high-income and low-income groups was 25%. The gap between the low-income and high-income groups was decomposed into its components. The decomposition analyses indicated that selected socio-demographic factors jointly explained a large proportion of the inequalities in unprotected sex among PWID. The selected predictor variables (age, education level, marital status, age of first engaging in unprotected sex, knowledge of partner’s HIV status, dropping out of school, age of first drug use, age of first injection, history of incarceration, access to harm reduction programs, HIV⁄AIDS knowledge, and methamphetamine use) together explained 47% (12 percentage points of the 25% gap) of the total inequality in unprotected sex, and the remaining 13 percentage points constituted the unexplained residual. Access to harm reduction programs made the largest contribution to the total inequality in unprotected sex among PWID. These results imply that the high-low disparity in unprotected sex could be reduced by only 47% even if the 2 groups became equivalent in all the covariates in the regression model. Moreover, HIV⁄AIDS knowledge and methamphetamine use accounted for about 13.5 and 7.5% of the total health inequality, respectively. The remaining gap was due to differences in the effects of the variables studied, as well as other factors that were not included in this study (unexplained components or coefficient effects). This may be attributed to factors correlated with economic status that were not included.

## DISCUSSION

In the present study, we estimated the absolute difference in unprotected sex in PWID in Tehran, comparing those with a low economic status to those with a high economic status, and identified sources of economic inequalities. The decomposition analysis showed that only about 47% of disparities in unprotected sex among PWID could be reduced by equalizing commonly observable characteristics in these 2 income groups. In this study, we found that PWID who had a low economic status were more likely to have unprotected sex than PWID who had a high economic status. These findings align with those of studies that have found lower socioeconomic status to be associated with HIV risk behaviors, such as unprotected sex, in PWID [24-27]. Many studies using regression models have shown a correlation between socioeconomic status and unprotected sex among PWID [25,28, 29]. The results of this study showed that access to harm reduction programs was a major contributor to economic inequalities in unprotected sex. Overall, these factors explained 22% (5.5 percentage points of the 25% gap) of the economic inequalities among PWID in Tehran. This is consistent with previous international studies [30,31]. These findings emphasize the importance of targeted prevention programs, such as harm reduction programs, for sexual risk behaviors. The results of this study showed that HIV⁄AIDS knowledge played an important role in economic inequalities in unprotected sex, in accordance with a previous study conducted in China and the results of a review of international evidence [30,31]. Nazari et al. [26] found a negative association between HIV knowledge and unprotected sex, and reported that higher HIV knowledge levels could reduce unprotected sex among PWID. Hajebi et al. [31] indicated that adding a skill-based HIV prevention psychoeducation program to NSPs effectively reduced high-risk sexual behaviors among the clients of 2 drop-in centers in Tehran. Our findings suggest that striving to promote HIV knowledge in lower income groups might be effective in buffering the effects of economic inequality. Thus, HIV prevention programs should strongly focus on promoting HIV knowledge among their clients. Policymakers should pay more attention to implementing and expanding education programs on HIV at drop-in centers for PWID. Moreover, based on the results of our study, the contribution of economic inequalities to sexual risk behaviors was partially explained by other factors. This is consistent with the results of a previous study conducted in 21 countries in sub-Saharan Africa [17]. Using the BO approach, we found that socio-demographic characteristics partly explained the economic inequality in high-risk sexual behaviors among PWID in Tehran. Our findings showed that participants’ educational level could explain inequalities in sexual risk behaviors. This is in accordance with a recent study by Chikovani et al. [13] that demonstrated that a higher education level was negatively associated with sexual risk behaviors in Georgia.

There were several limitations to this study. The first major limitation was its cross-sectional design, because it was not possible for us to investigate causal relationships directly. Therefore, a longitudinal study is required. Furthermore, our data were based on participants’ self-reports, making our findings potentially subject to recall and social desirability bias [33]. Third, the sample was not random, and participants were recruited using snowball sampling techniques, which may have biased the sample because of the size of participants’ social networks and homophily in recruitment patterns. Caution is therefore necessary in generalizing our results to all PWIDs living in Iran. The determinants of economic inequalities in HIV risk behaviors are poorly understood. Sociodemographic factors have been found to contribute to differences in HIV risk behaviors between low- and high-income groups [17].

In conclusion, the contribution of economic inequalities to high-risk sexual behaviors was primarily explained by the differential effects of access to NSPs and HIV knowledge among PWID. Increasing NSP coverage and improving HIV knowledge are therefore essential for efforts to eliminate inequalities in HIV risk behaviors among PWID.

## Figures and Tables

**Table 1.** Overall characteristics of the sample of PWID, Tehran, 2016 (n=500)

Characteristics	n (%)
Age (mean ± SD, yr)	33.45±9.7
Education	
Completed high school	318 (63.6)
High school or less	182 (36.4)
Living status	
Homeless	208 (41.6)
Stable housing	292 (58.4)
Monthly income (USD)	
<150	196 (39.2)
≥150	304 (60.8)
Age of first drug use	
<25	260 (52.0)
≥25	240 (48.0)
Age of first drug injection	
<25	245 (49.0)
≥25	255 (51.0)
Dropped out of school	
Yes	210 (42.0)
No	290 (58.0)
Access to harm reduction programs	
Yes	227 (45.4)
No	273 (54.6)
HIV knowledge	
High	355 (71.0)
Low	145 (29.0)
Employment status	
Unemployed	352 (70.4)
Employed	148 (29.6)
Marital status	
Not married	307 (61.4)
Married	193 (38.6)
Unprotected sex in the past 6 mo	200 (40.2)

PWID, people who inject drugs; SD, standard deviation; USD, US dollar; HIV, human immunodeficiency virus.

**Table 2.** Factors associated with unprotected sex in subgroups of PWID defined by economic status

	High-income	Low-income
Age	1.53 (1.23, 1.74)	1.14 (0.72, 1.80)
Education	1.00 (1.54, 1.34)	0.92 (0.52, 1.80)
Living status	1.48 (1.26, 1.84)	2.91 (1.88, 4.50)
Marital status	0.44 (0.18, 1.60)	0.46 (0.74, 2.87)
Methamphetamine use	1.15 (0.74, 1.27)	1.20 (0.71, 1.90)
Dropped out of school	1.48 (0.90, 2.44)	1.23 (0.71, 1.92)
Age of first drug use	1.12 (0.72, 1.80)	1.42 (0.74, 2.85)
Age of first unprotected sex	1.10 (0.51, 2.02)	1.20 (0.68, 2.07)
Age of first drug injection	0.31 (0.05, 2.10)	0.92 (0.52, 1.74)
History of incarceration	1.22 (0.78, 1.93)	0.76 (0.48, 1.31)
Knowledge of partner’s HIV status	1.34 (0.63, 2.78)	2.10 (0.91, 3.54)
HIV knowledge	1.60 (1.26, 2.84)	2.85 (1.78, 4.40)
Access to harm reduction programs	2.70 (2.05, 3.74)	1.30 (0.40, 2.78)

Values are presented as odds ratio (95% confidence interval). PWID, people who inject drugs; HIV, human immunodeficiency virus.

**Table 3.** Decomposition analysis of the contribution of socio-demographic factors to economic inequalities in unprotected sex

Variables	Coefficient	95% CI	Contribution (%)
Prevalence in high-income group	0.152	0.640, 0.150^*^	
Prevalence in low-income group	0.402	0.162, 0.310^*^	
Differences	0.250	0.180, 0.033^*^	100.00
Differences in characteristics	0.123	0.082, -0.153	47.00
Age	0.001	-0.005, 0.005	-0.12
Educational level	0.019	0.002, 0.036	6.42
Marital status	0.008	0.700, -2.300	2.29
Age of first unprotected sex	-0.003	-0.007, 0.001	-1.04
Knowledge of partner’s HIV status	-0.004	-0.012, 0.004	-0.54
Dropping out of school	<0.001	-0.005, 0.005	0.90
Age of first drug use	0.003	-0.007, 0.014	1.34
Age of first injection	-0.001	-0.009, 0.006	0.54
History of incarceration	-0.005	-0.020, 0.010	-2.95
Access to harm reduction programs	0.055	0.032, 0.078	21.40
HIV⁄AIDS knowledge	0.040	0.023, 0.058	13.56
Methamphetamine use	0.018	0.007, 0.029	7.50
HIV testing	-0.001	-0.003, 0.001	-0.22
Due to difference in coefficients (odds ratios)	0.134	0.068, 0.201	53.00
Age	0.006	-0.007, 0.019	2.36
Educational level	-0.001	-0.011, 0.010^*^	-0.21
Marital status	0.003	-0.019, 0.025	1.20
Dropping out of school	0.002	-0.012, 0.017	0.87
Age of first drug use	0.220	-0.019, 0.063	8.43
Age of first injection	-0.001	-0.011, 0.010	0.21
Age of first unprotected sex	-0.013	-0.041, 0.015	-5.20
Access to harm reduction programs	-0.067	-0.215, 0.081	-26.60
HIV⁄AIDS knowledge	-0.040	-0.243, 0.145	-18.89
Knowing partner’s HIV status	0.110	-0.036, 0.256	-42.70
Methamphetamine use	-0.104	-0.224, 0.016	-40.35
History of incarceration	-0.001	-0.011, 0.010	0.23
HIV testing	0.011	-0.082, 0.105	4.37
Constant	0.70	0.01, 1.50	75.70

CI, confidence interval; HIV, human immunodeficiency virus; AIDS, acquired immune deficiency syndrome.

* p<0.05.
